# A mixed-methods study on impact of active case finding on pulmonary tuberculosis treatment outcomes in India

**DOI:** 10.1186/s13690-024-01326-0

**Published:** 2024-06-20

**Authors:** Akshat P. Shah, Jigna D. Dave, Mohit N. Makwana, Mihir P. Rupani, Immad A. Shah

**Affiliations:** 1 Department of Community Medicine, Government Medical College Bhavnagar (Maharaja Krishnakumarsinhji Bhavnagar University), Near ST Bus Stand, Jail Road, Bhavnagar, Gujarat 364001 India; 2 Department of Respiratory Medicine, Government Medical College Bhavnagar (Maharaja Krishnakumarsinhji Bhavnagar University), Jail Road, Bhavnagar, Gujarat 364001 India; 3 Department of Community and Family Medicine, All India Institute of Medical Sciences (AIIMS), Khanderi, Parapipaliya, Rajkot, Gujarat 360006 India; 4 Clinical Epidemiology (Division of Health Sciences), ICMR – National Institute of Occupational Health (NIOH), Indian Council of Medical Research (ICMR), Meghaninagar, Near Raksha Shakti University, Ahmedabad, Gujarat 380016 India; 5 Division of Agricultural Statistics, Sher-e-Kashmir University of Agricultural Sciences & Technology of Kashmir, Jammu & Kashmir, Srinagar, 190025 India

**Keywords:** Active case finding, Passive case finding, TB treatment outcomes, National TB elimination program, TB program functionaries, India

## Abstract

**Background:**

Tuberculosis (TB) remains a significant public health burden in India, with elimination targets set for 2025. Active case finding (ACF) is crucial for improving TB case detection rates, although conclusive evidence of its association with treatment outcomes is lacking. Our study aims to investigate the impact of ACF on successful TB treatment outcomes among pulmonary TB patients in Gujarat, India, and explore why ACF positively impacts these outcomes.

**Methods:**

We conducted a retrospective cohort analysis in Gujarat, India, including 1,638 pulmonary TB cases identified through ACF and 80,957 cases through passive case finding (PCF) from January 2019 to December 2020. Generalized logistic mixed-model compared treatment outcomes between the ACF and PCF groups. Additionally, in-depth interviews were conducted with 11 TB program functionaries to explore their perceptions of ACF and its impact on TB treatment outcomes.

**Results:**

Our analysis revealed that patients diagnosed through ACF exhibited 1.4 times higher odds of successful treatment outcomes compared to those identified through PCF. Program functionaries emphasized that ACF enhances case detection rates and enables early detection and prompt treatment initiation. This early intervention facilitates faster sputum conversion and helps reduce the infectious period, thereby improving treatment outcomes. Functionaries highlighted that ACF identifies TB cases that might otherwise be missed, ensuring timely and appropriate treatment.

**Conclusion:**

ACF significantly improves TB treatment outcomes in Gujarat, India. The mixed-methods analysis demonstrates a positive association between ACF and successful TB treatment, with early detection and prompt treatment initiation being key factors. Insights from TB program functionaries underscore the importance of ACF in ensuring timely diagnosis and treatment, which are critical for better treatment outcomes. Expanding ACF initiatives, especially among hard-to-reach populations, can further enhance TB control efforts. Future research should focus on optimizing ACF strategies and integrating additional interventions to sustain and improve TB treatment outcomes.

**Supplementary Information:**

The online version contains supplementary material available at 10.1186/s13690-024-01326-0.

**Table:** 

Text box 1. Contributions to the literature
• Our study underscores active case finding’s (ACF’s) significant role in improving TB treatment outcomes in India, offering evidence-based insights.
• We identify district-level variability in treatment outcomes and consistent predictors across settings, enhancing contextual understanding.
• Insights into ACF mechanisms emphasize its importance in early case detection and treatment initiation, critical for reducing TB morbidity and mortality.
• Qualitative perspectives provide practical strategies to optimize ACF effectiveness, addressing complex TB challenges in resource-limited settings.

## Introduction

Tuberculosis (TB) remains a formidable public health challenge worldwide, with the World Health Organization (WHO) estimating 10.6 million new cases reported globally in 2022 [1]. India, as one of the most populous countries, shoulders a significant burden of this disease, reporting 2.8 million incident cases of TB in the year 2022, constituting nearly 27% of the global caseload [1, 2]. In fact, India consistently reports the highest number of TB cases globally [1–4]. Within India, the state of Gujarat recorded approximately 1.5 lakh notified TB cases in 2021 [2]. This high prevalence of TB underscores the urgent need for effective strategies to control and eliminate the disease [5]. One of these strategies is active case finding (ACF), a proactive approach involving systematic screening of the population through house-to-house visits conducted by healthcare staff, a part of national strategic plan of TB elimination in India [5]. In the year 2022, 48,329 TB cases (2.5%) were diagnosed through ACF in India [2].

The World Health Organization (WHO) advises conducting TB screening for individuals in close contact with TB patients, those living with HIV, as well as various vulnerable groups such as miners, prisoners, migrants, and indigenous populations [6]. Globally, evidence exists to understand the efficacy of ACF compared to passive case finding (PCF) in increasing case notifications and detecting undetected cases [7, 8]. Importantly, evidence suggests that the efficacy of ACF in detecting new cases is particularly pronounced in developing countries, as compared to developed ones [9–14]. For instance, in the Philippines, ACF detected high rates of TB, whereas in Indonesia, no cases were detected through ACF [15, 16]. However, among pregnant women, the number of new TB cases diagnosed through ACF has been reported to be low [17].

In India, the national TB program introduced ACF in 2017 with a focus on several key populations [18]. India has set an ambitious goal to eliminate TB by the year 2025, 5 years ahead of the global targets [5, 18, 19]. Previous studies in India have reported varying rates of TB positivity through ACF, ranging from as low as 0.17% among migrant workers to 17% overall [19, 20]. Primarily, studies have emphasized the increase in TB case detection yields through ACF [21]. Additionally, research has focused on documenting the outcomes of contact investigations, revealing rates as high as 5% in southern India and 1.5% in remote tribal areas [19, 22].

ACF plays a multifaceted role in curbing the TB epidemic: it not only reduces disease transmission by shortening the infectious period of patients but also contributes to earlier diagnosis and treatment, ultimately enhancing treatment outcomes [23–25]. Despite the importance of ACF, existing systematic reviews and meta-analyses fail to adequately address its association with TB treatment outcomes [26]. For instance, while one meta-analysis found no difference in treatment success between ACF and PCF groups [27], studies outside India have also reported conflicting results [28, 29]. Moreover, within India, there is limited and inconsistent evidence on the impact of ACF on TB treatment outcomes [30–33]. Such inconsistencies underscore the critical need for further investigation. Thus, our study aims to bridge this gap by establishing the association between ACF and TB treatment outcomes while also exploring why ACF positively impacts these outcomes.

## Methods

### Study design and duration

Our study employed an explanatory mixed-methods approach, integrating a retrospective cohort study with qualitative interviews. We aimed to explore the impact of active case finding (ACF) on TB treatment outcomes and gather perspectives from key stakeholders regarding the mechanisms through which ACF contributes to the improvement of TB treatment outcomes. Data collection for the quantitative component spanned September 2022. Qualitative interviews with key stakeholders from the National TB Elimination Program (NTEP) were conducted between January and April 2023, guided by a comprehensive topic guide.

### Study setting

Our study used state-level data from Gujarat, India. Gujarat, with a population of 60,439,692 and a literacy rate of 78% according to the 2011 Census, presents a diverse and significant setting for TB control efforts [34]. The state reported 104,696 TB cases in the public sector and 54,462 in the private sector in 2019 [35]. With approval from the State TB Cell of Gujarat, we accessed data on ACF and PCF for 2019 and 2020 through the Nikshay online portal. Initially, ACF in India was conducted thrice yearly for 15 days each in vulnerable populations [18]. In Gujarat, ACF surveys are now held every 2nd and 4^th^ Tuesday monthly, with flexibility to increase frequency. Health workers follow a protocol for symptom screening and testing using smear microscopy, X-rays, and nucleic acid amplification tests [18, 36]. Gujarat, with 33 districts, eight municipal corporations, and three union territories, has made significant strides in TB management, reporting the lowest TB prevalence reported in a recent nationwide survey [37]. The state has a treatment success rate of 89%, low loss to follow-up rates at 1.8% and a treatment failure rate of 0.7% [2]. Nearly 50% of ACF-diagnosed cases did not present typical symptoms but showed radiological evidence of TB, suggesting early detection [2]. Gujarat’s ACF program targets approximately 6.2 million individuals, including those in de-addiction centers and with mental health conditions, enhancing TB detection efforts [2].

### Study population

#### Quantitative

We enrolled 82,595 eligible patients diagnosed with pulmonary TB from 249,966 notified cases between January 2019 and December 2020 across 39 diagnosing districts in Gujarat (see Supplementary Table 1 in Additional file 1 for detailed distribution across different diagnosing districts). The exposure group consisted of 1,638 patients diagnosed through ACF, while 80,957 patients diagnosed through PCF served as the unexposed group. We applied strict exclusion criteria to maintain data quality [38].

#### Qualitative

We employed a purposive sampling strategy to ensure diverse perspectives from various TB program sectors, with participants’ experience ranging from 2 to 25 years. We conducted 11 interviews with key stakeholders, including the district and city TB officers, a senior treatment supervisor, an additional director from the State TB Training and Demonstration Center (STDC) in Ahmedabad, WHO consultants from the TB cell in Gandhinagar, the head of the TB laboratory at STDC in Ahmedabad, and an epidemiologist from STDC in Ahmedabad. Interview durations ranged from 5 to 22 min. Saturation of responses was monitored throughout, with two additional interviews conducted to confirm that saturation had been reached.

### Study variables (quantitative)

#### Outcome variable

Successful treatment outcomes encompass patients categorized as “cured” (those with a negative sputum at the end of treatment) and those who have “completed treatment” (patients who have finished the full course of treatment without any radiological or clinical deterioration) [36]. Conversely, unsuccessful outcomes include individuals categorized as “loss to follow-up” (patients who discontinued treatment for at least one consecutive month), “treatment failure” (patients with sputum positivity at the end of treatment), and “died” (patients who passed away while undergoing treatment) [36].

#### Exposure variable

Patients were categorized based on their diagnostic method: ACF (exposed group) and PCF (unexposed group).

#### Confounding variable

We considered several potential confounding variables, including age, gender, sputum positivity, HIV status, and diabetes status.

### Data collection

#### Quantitative

Data were collected from the Nikshay online portal, managed by the State TB Training and Demonstration Centre (STDC) in Ahmedabad, Gujarat. Detailed district-wise Excel spreadsheets were used, containing information on patients diagnosed through ACF. These were cross-referenced with Nikshay IDs from the notification register to distinguish between ACF and PCF cases. Permission was obtained before the retrospective data collection process. The dataset included age, gender, address, HIV status, diabetes status, TB site, sputum positivity, and treatment outcomes for both ACF and PCF cases, covering Gujarat from January 1, 2019, to December 31, 2020.

#### Qualitative

In-depth interviews were conducted by the principal investigator and experienced co-investigators using a purposive sampling approach. Interviewees were selected for their ability to offer diverse insights relevant to comparing treatment outcomes between TB patients identified through ACF and PCF. All interviews were conducted face-to-face in a conducive environment, with flexibility in scheduling to accommodate participants’ availability. One initial refusal was resolved by including an alternative participant. To enhance data validity and reliability, the interview guide (see Additional file 2) was pilot tested among study authors. The data collection process was meticulously documented, including informed consent and audio recordings.

### Statistical analysis

#### Quantitative

Data analysis was performed using RStudio version 4.3.3, with a significance threshold of *p* < 0.05. Generalized logistic mixed-effects models (GLMM) were used to estimate both random and fixed effects, employing the glmer() function from the {lme4} package in RStudio. The model included fixed effects (ACF, age, gender, HIV status, diabetes status, and sputum result) and random effects (diagnosing district), accounting for clustering by district. The model used a binomial family with a logit link function, suitable for binary outcome data. The intra-cluster correlation coefficient (ρ) was calculated using the clus.rho() command from the {fishmethods} package in RStudio to measure clustering resemblance within groups. Bootstrap analysis was performed using the boot() function from the {boot} package in RStudio to estimate the uncertainty of predictor coefficients.

#### Qualitative

All interviews were conducted in Gujarati for cultural sensitivity and participant engagement, then transcribed into English. The transcripts were documented in Microsoft Word (see Additional file 3), facilitating efficient data management and analysis. Investigators A.S. and M.M. assigned codes to the transcriptions, which were compiled into a Microsoft Excel sheet. Codes were organized into meaningful categories using an inductive approach, allowing themes to emerge directly from the data. Regular discussions and reviews among investigators M.R., J.D., and M.M. ensured the trustworthiness and reliability of the analysis. Investigator A.S. established a final codebook (see Additional file 4) to provide a standardized framework for subsequent data analysis and interpretation.

## Results

### Quantitative

#### Selection of study participants

**Fig. 1:**
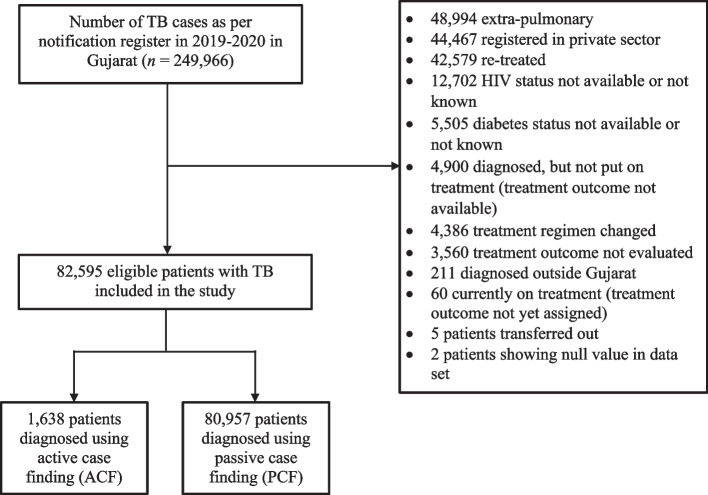
Selection of patients notified with pulmonary TB in the public sector in Gujarat during 2019–2020

A data of total 249,966 was collected, of which 82,595 eligible patient data was included in the further analysis (Fig. 1). Out of the total eligible sample size, 1638 were diagnosed through ACF and the remaining 80,957 were diagnosed through PCF.

#### Characteristics of study participants

ACF patients, with a median age of 43 years (IQR: 29–58), were older than PCF patients, whose median age was 36 years (IQR: 25–52) (*p* < 0.001) (Table 1). While no significant gender difference was observed between the groups (ACF: 34% female, PCF: 34%, *p* = 0.738), notable disparities were found in HIV positivity (ACF: 0.4%, PCF: 3%, *p* < 0.001) and diabetes prevalence (ACF: 5%, PCF: 6%, *p* = 0.03). Additionally, sputum-positive TB cases were less frequent in ACF (55%) compared to PCF (64%) (*p* < 0.001). Treatment success rates were higher in ACF (94%) than in PCF (91%) (*p* < 0.001).

**Table 1:** Characteristics of pulmonary TB patients diagnosed through ACF vs. PCF during 2019–2020 in Gujarat (*n* = 82,595)

**Characteristics**	**ACF (*n* = 1638)**	**PCF (*n* = 80,957)**	***P*-value**
	**n (%) or median (IQR)**	**n (%) or median (IQR)**	
Age (years)	43 (29–58)	36 (25–52)	< 0.001^*^
Male gender	1082 (66)	53,802 (67)	0.733^#^
HIV positive	6 (0.4)	2147 (3)	< 0.001^#^
Diabetic	81 (5)	5063 (6)	0.03^#^
Sputum positive TB	906 (55)	51,832 (64)	< 0.001^#^
Treatment outcomes			
Successful			< 0.001^#^
Cured	849 (52)	45,351 (56)	
Treatment completed	695 (42)	28,679 (35)	
Unsuccessful			
Died	58 (4)	4510 (6)	
Treatment failure	11 (0.7)	763 (0.9)	
Lost to follow up	25 (1.5)	1654 (2)	

*IQR* Interquartile range, *HIV* Human immunodeficiency virus, *TB* Tuberculosis

^*^Median test ^#^Pearson’s chi-square test

#### Association of ACF with successful TB treatment outcomes

On our generalized logistic mixed-effects model analysis, we found that individuals identified through ACF had 1.4 times higher odds (95% CI: 1.12–1.73, *p* = 0.002) of achieving successful TB treatment outcomes compared to those identified through PCF (Table 2). Furthermore, Each additional year of age was associated with a 3% decrease in the odds of successful TB treatment (adjusted OR: 0.973, 95% CI: 0.971–0.974, *p* < 0.001). Male individuals exhibited a 30% decrease in odds compared to female individuals (adjusted OR: 0.70, 95% CI: 0.66–0.74, *p* < 0.001). HIV-positive individuals demonstrated a 76% lower odds of successful TB treatment compared to HIV-negative individuals (adjusted OR 0.24, 95% CI: 0.21–0.26, *p* < 0.001). Additionally, individuals with sputum-positive TB had a 32% lower odds of successful TB treatment compared to those with sputum-negative results (adjusted OR: 0.68, 95% CI: 0.65–0.72, *p* < 0.001). Interestingly, diabetic status did not show a significant association with treatment success (adjusted OR: 1.01, 95% CI: 0.92–1.11, *p* = 0.824).

**Table 2:** Generalized logistic mixed-effects model analysis for association of ACF and other predictors with successful TB treatment outcomes across 39 diagnosing districts during 2019–20 in state of Gujarat (*n* = 82,595)

**Variables**	**Fixed effects**		**Random effects (diagnosing district)**
	**Adjusted OR (95% CI)**	***p*-value**	**Variance**
Intercept	63 (55–72)	< 0.001	0.063
Active case finding (ACF)	1.4 (1.12–1.73)	0.002	-
Age (years)	0.973 (0.971–0.974)	< 0.001	-
Male gender	0.70 (0.66–0.74)	< 0.001	-
HIV positive	0.24 (0.21–0.26)	< 0.001	-
Diabetic	1.01 (0.92–1.11)	0.824	-
Sputum positive TB	0.68 (0.65–0.72)	< 0.001	-

Moreover, our analysis accounted for variability across districts, revealing a variance of 0.063 attributed to diagnosing facility district, indicating significant variations in successful TB treatment outcomes between different districts. The intra-cluster correlation coefficient (ICC) for the exposure group (ACF vs. PCF) was found to be 0.0101, suggesting a weak resemblance of the units within each particular group (see Additional file 5). Furthermore, despite considering multiple districts in our study, the coefficients for all predictors remained consistent, as observed from the bootstrap analysis (see Additional file 5).

### Qualitative

**Fig. 2:**
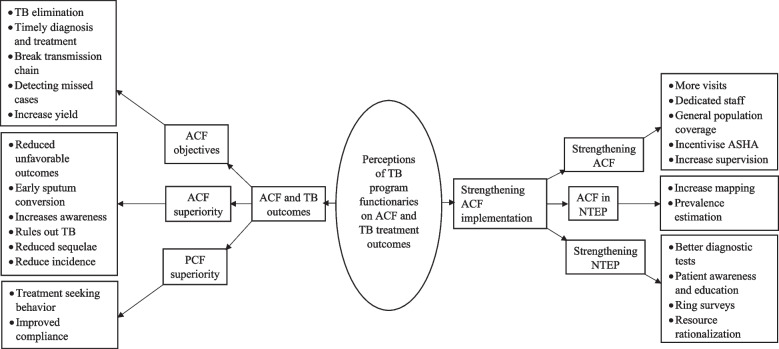
Perceptions of TB program functionaries on active case finding (ACF) and TB treatment outcomes during January-April 2023 in Gujarat

The median (IQR) years of experience for the 11 participants in the in-depth interviews were 12 (2–25) years. Among the participants, one was a female. Analysis of the codebook resulted in the identification of two fundamental themes: ‘ACF and TB outcomes’ and ‘Strengthening ACF implementation’ (see Fig. 2 and read Additional file 4 for description of each code).

#### ACF and TB outcomes

Program functionaries highlighted the pivotal role of ACF within the national TB program. ACF was emphasized for its effectiveness in augmenting case detection rates, identifying previously undetected TB cases, and enabling early detection and prompt initiation of TB treatment. This approach helps achieve quicker non-infectious status among patients, facilitates early sputum conversion, and breaks the chain of TB transmission from asymptomatic primary cases to their close contacts. Consequently, ACF reduces the incidence of new cases and prevents the progression of the disease in untreated asymptomatic cases. Within the framework of the national TB program, ACF was viewed as a superior strategy compared to PCF.

*“There are research studies that show that the patients getting diagnosed, may likely to get delayed up to 15-20 days as well as they visit around 1-7 facilities before actually diagnosing the TB cases. So, this ACF model is a good implementation to have an early case detection and that is why we are going house-to-house to detect cases early. So overall, ACF is great tool to detect those missing cases, as well as kind of early case detection.”* (TB program functionary, 9 years of work experience)

#### Strengthening ACF implementation

Experts highlighted several avenues for strengthening the implementation of ACF. These include increasing field visits, deploying dedicated staff for comprehensive surveillance, incentivizing healthcare workers, and intensifying supervision. Additional strategies to strengthen national TB program include improving diagnostic capabilities, raising awareness, and optimizing resource allocation. Experts also stressed the importance of screening for latent tuberculosis infection (LTBI) within communities, aiming to identify individuals at risk and facilitate early intervention through preventive treatment. In resource-constrained settings, prioritizing secondary prevention over primary prevention was seen as more practical for TB elimination due to factors such as large family sizes, overcrowding, poor nutrition, limited awareness, restricted healthcare access, and neglect.

*“We need to improve and strengthen the supervision. We are training the supervisory staff, so that they can check the quality and number of sputum samples collected by the field worker. Also, the field worker needs to be trained on how to collect the sputum. How to counsel the suspect and his family. It will improve the overall outcome of the program.”* (TB program functionary, 25 years of work experience).

*“Our aim is not only increasing the number of case detection, but also parallelly rule out TB in the same community. Those ruled out, should be put on preventive treatment, by checking for LTBI [latent tuberculosis infection] positivity. So, the objective of the intervention is not only case detection, but also to prevent LTBI.”* (TB program functionary, 9 years of work experience).

## Discussion

### Summary and brief explanation of findings

Our study underscores the significant impact of active case finding (ACF) on improving TB treatment outcomes in Gujarat. ACF, accounting for 2% of TB cases in our study, aligns with the national average of 2.5% in India [18], signifying its important role in improving TB treatment outcomes. Program functionaries emphasized the urgency to intensify ACF practices to enhance early case detection and prompt initiation of TB treatment, achieving quicker non-infectious status, and facilitate early sputum conversion, thereby reducing the spread of TB [23].

The favorable treatment outcomes observed in Gujarat can be attributed to several factors, including the state’s diligent TB care and support infrastructure, characterized by the lowest TB prevalence, a success rate of 89%, low loss to follow-up rates at 1.8%, and a treatment failure rate of 0.7% [2, 37]. Notably, the implementation of bi-monthly ACF activities, particularly on Tuesdays, likely played a significant role in these outcomes. The rigorous execution of these ACF practices facilitates early TB case detection and prompt treatment initiation, contributing to the observed improvements in treatment outcomes [39].

Moreover, our analysis revealed significant variability in successful TB treatment outcomes between different districts, with a variance of 0.063 attributed to diagnosing district. This underscores the importance of considering contextual factors and district-level variations in TB management strategies. While there may be some clustering of treatment outcomes within ACF groups, individual-level factors remain influential, as indicated by the ICC for ACF (0.0101). Despite this variability, the coefficients for all predictors remained consistent across districts, as observed from the bootstrap analysis. This suggests that the effects of predictors on treatment outcomes are robust and reliable across diverse settings, emphasizing the need for tailored interventions to address district-specific challenges while leveraging consistent predictors for TB treatment success.

### ACF and TB treatment outcomes

Our study demonstrated that individuals diagnosed with TB through ACF had 1.4 times higher odds of successful treatment outcomes compared to those identified through passive case finding (PCF). In contrast to our findings, a study in Haridwar reported a 2.6 times higher risk of unsuccessful TB treatment outcomes associated with ACF compared to PCF, albeit on a small sample size [30]. Similarly, another study in the same district, albeit also with a small sample size, reported a much lower treatment success rate of 64% compared to the 94% reported in our study [32]. A study in South Delhi, again with a limited sample size, did not find a significant association between ACF and TB treatment outcomes, with treatment success rates of 75% for ACF and 82% for PCF [33]. Furthermore, a larger study across India, although not showing a significant association between ACF and treatment outcomes (ACF 90% vs. PCF 87% successful TB treatment outcomes), suggested a 17% lower chance of unfavorable TB treatment outcomes with ACF, underscoring its potential advantage [31]. Finally, a study among tribal populations in Madhya Pradesh with a substantial sample size found improvements in TB treatment outcomes due to ACF [19]. Considering the limitations of previous studies with small sample sizes and the trend of well-conducted studies favoring ACF in improving TB treatment outcomes, our study’s findings on a large sample size unquestionably underscore the value of ACF in enhancing TB treatment outcomes among pulmonary TB patients.

When comparing our study results with international evidence, we observed higher treatment success rates in the ACF group compared with the PCF group, while studies in Myanmar, Ethiopia, Nigeria, and South Africa, including a systematic review, reported similar treatment success rates in these groups [14, 25, 27–29]. Additionally, most existing studies have reported high initial default rates and treatment delays among patients diagnosed through community surveys [16, 25, 27, 30, 32, 40], although one study noted a decrease in the initial default rate due to ACF [19]. However, our study did not specifically investigate this effect as it focused on TB patients already on treatment. Lastly, exploring the impact of community-based ACF initiatives on TB treatment outcomes in trial settings could provide valuable insights into the effectiveness of ACF in improving TB treatment outcomes [41].

The observed enhancement in treatment outcomes associated with ACF in our study can be attributed to several potential mechanisms. Firstly, ACF facilitates the early detection of TB cases within the community [13, 21], enabling prompt initiation of treatment [12]. By actively screening individuals who may not present with typical TB symptoms but are still infectious, ACF minimizes delays in diagnosis and treatment initiation [19, 42, 43], crucial factors in preventing disease progression and transmission [6, 8, 25, 39, 44], ultimately reducing morbidity, mortality, and the overall burden of TB. Additionally, ACF allows for the identification of TB cases at an earlier stage of the disease, when treatment is more effective and complications are less likely to occur [21, 25, 42, 44, 45]. This early detection and treatment initiation may contribute to a higher proportion of cases achieving cure status, thereby improving treatment outcomes.

Furthermore, ACF interventions often involve comprehensive patient management and support services, including counseling, adherence support, and monitoring throughout the treatment process [33]. By engaging TB patients early in their care journey, ACF programs can address barriers to treatment adherence and facilitate patient-centered care, which are known determinants of treatment success [29, 33]. Moreover, ACF activities may contribute to increased awareness and knowledge about TB within the community, leading to reduced stigma, improved health-seeking behavior, and earlier presentation to healthcare facilities among individuals with TB symptoms [19, 24, 45, 46]. These findings emphasize the critical role of ACF in achieving better TB treatment outcomes, underscoring the need for continued support and implementation of ACF initiatives.

However, it is important to recognize the potential disadvantages of ACF in TB control programs. ACF programs can be resource-intensive, requiring significant financial and human resources for community outreach, screening, and follow-up [47, 48]. Additionally, the higher costs associated with ACF interventions compared to traditional PCF approaches can strain already limited healthcare budgets, especially in resource-constrained settings [42, 49–51]. To enhance the efficiency and effectiveness of ACF activities in India, two promising approaches can be considered: linking ACF activities with TB preventive treatment for household contacts [52, 53] and integrating cost-efficient artificial intelligence (AI) tools into chest X-ray screening for TB [54–57]. These approaches offer novel methods of secondary prevention, targeting both latent TB and asymptomatic TB cases, and could help mitigate some of the resource challenges associated with ACF, particularly in vulnerable populations [58].

### Secondary findings

Our analysis identified several significant predictors of favorable TB treatment outcomes. As individuals age, there might be an increased risk of unsuccessful TB treatment outcomes, including death and loss to follow-up, possibly due to challenges in treatment adherence in older populations [59]. Females demonstrated significantly higher treatment success rates compared to males, in contrast to other studies [60–63], possibly influenced by factors such as addiction prevalence and hormonal differences. Additionally, sputum negativity and HIV-negative status were associated with substantially higher treatment success rates, corroborating previous research findings [60, 61, 64–66]. Notably, our study contributes to a deeper understanding of these factors, overcoming limitations observed in prior studies. These findings underscore the critical role of targeted interventions and comprehensive care in improving TB treatment outcomes.

### Qualitative findings

In our qualitative analysis, TB program functionaries emphasized the critical role of ACF in improving TB treatment outcomes. This aligns with the quantitative findings, which highlighted the significance of ACF in enhancing treatment success rates. Program functionaries recommend increasing field visits, deploying dedicated staff, and incentivizing healthcare workers to maximize the effectiveness of ACF. By focusing on ACF and its direct impact on TB treatment outcomes, our qualitative findings provide a comprehensive perspective on the effectiveness of ACF strategies. This emphasizes the need for continued efforts to enhance ACF practices to improve TB treatment outcomes in India.

### Strengths and limitations

Our study presents significant strengths, including a substantial and representative sample size facilitating a thorough comparison of TB treatment outcomes between active and passive case finding methods. The incorporation of a qualitative component through in-depth interviews provides novel insights and complements the quantitative findings, offering valuable context to the dynamics of active and passive case finding. Adherence to established guidelines for reporting, including STROBE for quantitative findings and COREQ for qualitative findings [67, 68], enhances the transparency and credibility of the study, bolstering its scientific validity. Moreover, the generalizability of our findings extends beyond the study region, informing TB management strategies across the broader Indian context. However, certain limitations exist, including the exclusion of drug-resistant cases and extrapulmonary TB, potentially limiting the comprehensiveness of the results. The brief duration of interviews may have restricted the depth of insights obtained from participants, and the lack of data on smoking status and occupation could introduce confounding factors. Additionally, the selection of study years based on data availability and the potential limitations in the representativeness of the qualitative sample pose challenges. To address these concerns, future studies could explore additional years to provide a more comprehensive understanding of the impact of ACF on TB treatment outcomes. Moreover, efforts should be made to enhance the depth and breadth of qualitative data collection, including a more comprehensive sampling strategy and further exploration of contextual factors to improve the validity and reliability of qualitative findings.

## Conclusions

Our study sheds light on the potential of active case finding (ACF) to contribute to improved TB treatment outcomes in India. Through a mixed-methods approach, we found evidence suggesting an association between ACF and successful TB treatment outcomes, underscoring its importance in the context of TB control efforts. The insights gleaned from our quantitative analysis, supplemented by qualitative perspectives from TB program functionaries, provide valuable contributions to the understanding of how ACF positively impacts TB treatment outcomes. Notably, our findings suggest that expanding ACF initiatives to vulnerable populations and intensifying efforts such as ring surveys and preventive treatment could further improve the TB control measures. Future studies could explore the combined effectiveness of ACF with other targeted interventions to maximize impact and accelerate progress in TB control. In conclusion, our findings underscore the importance of ACF as a valuable tool in the fight against TB in India. By leveraging targeted strategies and collaborative efforts, we can significantly improve TB treatment outcomes and work towards alleviating the burden of this global health challenge.

### Supplementary Information

Supplementary Material 1.

Supplementary Material 2.

Supplementary Material 3.

Supplementary Material 4.

Supplementary Material 5.

## Data Availability

The data that support the findings of the quantitative component of the study are available from the Gujarat State TB Cell but restrictions apply to the availability of these data, which were used under license for the current study, and so are not publicly available. Data are however available from the authors upon reasonable request and with permission of the Gujarat State TB Cell. All data generated or analyzed during the qualitative in-depth interviews of the study are included in this published article [and its supplementary information files].

## Abbreviations

ACF: Active Case Finding
ASHA: Accredited Social Health Activist
COREQ: COnsolidated criteria for REporting Qualitative research
HIV: Human Immunodeficiency Virus
ICMR: Indian Council of Medical Research
IQR: Interquartile Range
LTBI: Latent Tuberculosis Infection
NTEP: National Tuberculosis Elimination Program
PCF: Passive Case Finding
STDC: State Tuberculosis Training and Demonstration Centre
STROBE: Strengthening The Reporting of OBservational studies in Epidemiology
TB: Tuberculosis
WHO: World Health Organization
