# Supplementary material for: A mixed-methods study on impact of active case finding on pulmonary tuberculosis treatment outcomes in India
Source: Arch Public Health. 2024 Jun 20;82:92. doi: 10.1186/s13690-024-01326-0 (PMC11188491; doi:10.1186/s13690-024-01326-0)
Supplement: Supplementary file 1 — Supplementary Material 1. [file 13690_2024_1326_MOESM1_ESM.docx]

Supplementary Table #: Distribution of diagnosing facility for the pulmonary tuberculosis patients in the study (n=82815)

| **District/ Corporation/ Union Territory** | **Frequency** | **Percent** |
| --- | --- | --- |
| **Diagnosed In Gujarat** | | |
| Ahmedabad Municipal Corporation | 7040 | 8.5 |
| Dahod district | 6689 | 8.1 |
| Banaskantha district | 4901 | 5.9 |
| Surat Municipal Corporation | 4159 | 5.0 |
| Panchmahals district | 4138 | 5.0 |
| Kheda district | 3779 | 4.6 |
| Anand district | 3478 | 4.2 |
| Vadodara district | 3151 | 3.8 |
| Rajkot (corporation + district) | 2746 | 3.3 |
| Mahesana district | 2643 | 3.2 |
| Bharuch district | 2525 | 3.0 |
| Surat district | 2424 | 2.9 |
| Surendranagar district | 2401 | 2.9 |
| Mahisagar district | 2353 | 2.8 |
| Bhavnagar (corporation + district) | 2294 | 2.8 |
| Patan district | 2168 | 2.6 |
| Sabarkantha district | 2122 | 2.6 |
| Gandhinagar (corporation + district) | 1815 | 2.2 |
| Vadodara Municipal Corporation | 1791 | 2.2 |
| Chhotaudepur district | 1784 | 2.2 |
| Arvalli district | 1770 | 2.1 |
| Ahmadabad district | 1712 | 2.1 |
| Kachchh district | 1712 | 2.1 |
| Valsad district | 1664 | 2.0 |
| Jamnagar (corporation + district) | 1511 | 1.8 |
| Navsari district | 1412 | 1.7 |
| Narmada district | 1329 | 1.6 |
| Vyara district | 1258 | 1.5 |
| Amreli district | 1202 | 1.5 |
| Junagadh (corporation + district) | 1150 | 1.4 |
| Gir Somnath district | 1143 | 1.4 |
| Morbi district | 613 | .7 |
| Porbandar district | 534 | .6 |
| Botad district | 444 | .5 |
| Devbhumi Dwarka district | 412 | .5 |
| The Dangs district | 220 | .3 |
| Dadra & Nagar Haveli (Union territory) | 51 | .1 |
| Daman (Union territory) | 48 | .1 |
| Diu (Union territory) | 11 | .0 |
| **Total Diagnosed In Gujarat** | **82597** | **99.7** |
| **Diagnosed Outside Gujarat** | | |
| Nandurbar | 14 | .0 |
| Ganjam | 10 | .0 |
| Udaipur | 7 | .0 |
| Parel | 6 | .0 |
| Ajmer | 5 | .0 |
| Sultanpur | 5 | .0 |
| Alirajpur | 4 | .0 |
| Pratapgarh | 4 | .0 |
| Prayagraj | 4 | .0 |
| Rajsamand | 4 | .0 |
| Varanasi | 4 | .0 |
| Bhind | 3 | .0 |
| Buldana | 3 | .0 |
| Byculla | 3 | .0 |
| Dhule | 3 | .0 |
| Dhule MC | 3 | .0 |
| Gwalior | 3 | .0 |
| Jalgaon | 3 | .0 |
| Nagaur | 3 | .0 |
| Navi Mumbai | 3 | .0 |
| Sant Kabir Nagar | 3 | .0 |
| Siwan | 3 | .0 |
| Thane | 3 | .0 |
| Ayodhya | 2 | .0 |
| Bhubaneshwar MC | 2 | .0 |
| Dhar | 2 | .0 |
| Ghazipur | 2 | .0 |
| Hamirpur-Up | 2 | .0 |
| Jaunpur | 2 | .0 |
| Jhabua | 2 | .0 |
| Jodhpur | 2 | .0 |
| Kota | 2 | .0 |
| Mahendragarh | 2 | .0 |
| Moga | 2 | .0 |
| Moti Nagar | 2 | .0 |
| Satna | 2 | .0 |
| Sirohi | 2 | .0 |
| Udhamsingh Nagar | 2 | .0 |
| Ujjain | 2 | .0 |
| Akola | 1 | .0 |
| Aligarh | 1 | .0 |
| Amethi | 1 | .0 |
| Anantapur | 1 | .0 |
| Andheri West | 1 | .0 |
| Baleshwar | 1 | .0 |
| Banda | 1 | .0 |
| Barwani | 1 | .0 |
| Basti | 1 | .0 |
| Betul | 1 | .0 |
| Bhagalpur | 1 | .0 |
| Bhilwara | 1 | .0 |
| Bhiwandi Nizampur | 1 | .0 |
| Bijnor | 1 | .0 |
| Bikaner | 1 | .0 |
| Borivali | 1 | .0 |
| Bsa Chest Clinic | 1 | .0 |
| Buxar | 1 | .0 |
| Centenary | 1 | .0 |
| Central Chennai | 1 | .0 |
| Champawat | 1 | .0 |
| Chitrakoot | 1 | .0 |
| Dakshin Dinajpur | 1 | .0 |
| Dakshina Kannada | 1 | .0 |
| Deoria | 1 | .0 |
| Dewas | 1 | .0 |
| Diamond Harbour | 1 | .0 |
| Erode | 1 | .0 |
| Etawah | 1 | .0 |
| Fatehpur | 1 | .0 |
| Firozabad | 1 | .0 |
| Ghatkopar | 1 | .0 |
| Gondiya | 1 | .0 |
| GTB Chest Clinic | 1 | .0 |
| Hazaribagh | 1 | .0 |
| Hisar | 1 | .0 |
| Howrah | 1 | .0 |
| Indore | 1 | .0 |
| Jalgaon MC | 1 | .0 |
| Jalore | 1 | .0 |
| Jhandewalan | 1 | .0 |
| Jind | 1 | .0 |
| Kalyan Dombivli MC | 1 | .0 |
| Kandivali | 1 | .0 |
| Kanpur Nagar | 1 | .0 |
| Khandwa | 1 | .0 |
| Khargone | 1 | .0 |
| Kollam | 1 | .0 |
| Lakhisarai | 1 | .0 |
| Ln Chest Clinic | 1 | .0 |
| Lrs | 1 | .0 |
| Madhubani | 1 | .0 |
| Mainpuri | 1 | .0 |
| Malegaon MC | 1 | .0 |
| Mohali | 1 | .0 |
| Morena | 1 | .0 |
| Nagpur MC | 1 | .0 |
| Nalanda | 1 | .0 |
| Nayagarh | 1 | .0 |
| Neemuch | 1 | .0 |
| North 24 Parganas | 1 | .0 |
| Pashchim Champaran | 1 | .0 |
| Pimpri Chinchwad | 1 | .0 |
| Purba Bardhaman | 1 | .0 |
| Rae Bareli | 1 | .0 |
| Raigad | 1 | .0 |
| Raipur | 1 | .0 |
| Rewa | 1 | .0 |
| Rohtak | 1 | .0 |
| Rohtas | 1 | .0 |
| Sant Ravidas Nagar | 1 | .0 |
| Saran | 1 | .0 |
| Satara | 1 | .0 |
| Sawai Madhopur | 1 | .0 |
| Shahadra | 1 | .0 |
| Shajapur | 1 | .0 |
| Sheikhpura | 1 | .0 |
| Sikar | 1 | .0 |
| Sindhudurg | 1 | .0 |
| Sitamarhi | 1 | .0 |
| Thane MC | 1 | .0 |
| Vaishali | 1 | .0 |
| Vasai Virar | 1 | .0 |
| **Total diagnosed outside Gujarat** | **211** | **0.3** |
| **Grand Total** | **82815** | **100.0** |
