# Supplementary material for: A mixed-methods study on impact of active case finding on pulmonary tuberculosis treatment outcomes in India
Source: Arch Public Health. 2024 Jun 20;82:92. doi: 10.1186/s13690-024-01326-0 (PMC11188491; doi:10.1186/s13690-024-01326-0)
Supplement: Supplementary file 2 — Supplementary Material 2. [file 13690_2024_1326_MOESM2_ESM.docx]

**Interview guide:**

**(ઇન્ટરવ્યૂ માર્ગદર્શિકા)**

**Purpose of this discussion:**

**(આ ચર્ચાનો હેતુ)**

Greetings everyone. We have gathered here today to discuss about the practice of active case finding (ACF) and treatment outcomes in patients of pulmonary tuberculosis.

(દરેકને શુભેચ્છાઓ. આજે આપણે અહીં પલ્મોનરી ટ્યુબરક્યુલોસિસ (**ક્ષય**) ના દર્દીઓમાં સક્રિય કેસ શોધવાની (એસીએફ) અને સારવારના પરિણામો જાણવાની પધ્ધ્તીની ચર્ચા કરવા ભેગા થયા છીએ.)

**Briefing:**

This is a part of a study by government medical college, Bhavnagar, among stakeholders of NTEP. The research question which is to be answered is “whether there are better treatment outcomes by practicing ACF, for the patients of pulmonary TB, as compared to that by PCF?”

(આ સરકારી મેડિકલ કોલેજ, ભાવનગર દ્વારા NTEP ના હિસ્સેદારો વચ્ચેના અભ્યાસનો એક ભાગ છે. પીસીએફની સરખામણીમાં પલ્મોનરી ટીબીના દર્દીઓ માટે એસીએફની પ્રેક્ટિસ કરીને સારવારના વધુ સારા પરિણામો છે કે કેમ તે જવાબ આપવાનો છે?)

This is not to test your knowledge on TB as a disease, so feel free to contribute whatever you know on the topic. There is no right or wrong answer in this discussion. We are here to know the impact of ACF on the treatment outcomes of pulmonary TB as well as solutions for overcoming any hold-backs.

(આ એક રોગ તરીકે ટીબી પર તમારા જ્ જ્ઞાનની ચકાસણી કરવા માટે નથી, તેથી તમે આ વિષય પર જે પણ જાણો છો તેમાં નિસંકોચ યોગદાન આપો. આ ચર્ચામાં કોઈ સાચો કે ખોટો જવાબ નથી. પલ્મોનરી ટીબીના સારવારના પરિણામો પર એસીએફની અસર તેમજ કોઈપણ હોલ્ડ-બેકને દૂર કરવાના ઉપાયો જાણવા માટે અમે અહીં છીએ.)

It is expected that you take your time and give your honest, unbiased, and complete opinion on the question in discussion. All the information will be kept confidential and anything told by you will not be linked with your names and it will not be disclosed to anyone, so feel free to give your comments and give your detailed opinion rather than just yes or no.

(એવી અપેક્ષા રાખવામાં આવે છે કે તમે તમારો પુરતો સમય લઇ અને ચર્ચામાં પ્રશ્ન પર તમારો પ્રામાણિક, નિષ્પક્ષ અને સંપૂર્ણ અભિપ્રાય આપો. બધી માહિતી ગુપ્ત રાખવામાં આવશે અને તમારા દ્વારા જણાવવામાં આવેલી કોઈપણ બાબતો તમારા નામો સાથે જોડવામાં આવશે નહીં અને તે કોઈને પણ જાહેર કરવામાં આવશે નહીં, તેથી નિસંકોચ ફક્ત હા અથવા ના કરતાં તમારી ટિપ્પણીઓ અને તમારો વિગતવાર અભિપ્રાય આપવો.)

**Opening questions:**

1. What is your post as a TB program functionary?

(ટીબી પ્રોગ્રામ કાર્યકારી તરીકે તમારી પોસ્ટ શું છે?)

1. How many years of experience do you have?

(તમારી પાસે કેટલા વર્ષોનો અનુભવ છે?)

**Specific questions:**

1. What according to you is the status of ACF in TB program?

(તમારા મતે ટીબી પ્રોગ્રામમાં ACF ની સ્થિતિ શું છે?)

1. What is your opinion on the importance of ACF in improving treatment outcomes of the patients?

(ACF ના, સારવાર ના પરિનામો પર ના મહત્વ વિશે તમારા શુ અભિપ્રાય છે?)

1. What are your views regarding the ACF/PCF being superior to the other?

(એસીએફ/પીસીએફ અન્ય કરતા શ્રેષ્ઠ હોવા અંગે તમારા મંતવ્યો શું છે?)

(probe: why do you think so?) (તમને આવું કેમ લાગે છે?)

1. What do you think, can ACF/PCF affect treatment outcomes of TB?

(તમને શું લાગે છે, ACF/PCF ટીબીના સારવારના પરિણામોને અસર કરી શકે છે?)

(probe: how?) (કેવી રીતે?)

1. What is your opinion on the importance of intensification of ACF as one of the interventions for achieving elimination of TB?

( ટીબી નાબૂદી માટે એક હસ્તક્ષેપ તરીકે એસીએફની તીવ્રતાના મહત્વ પર તમારો અભિપ્રાય શું છે?)

1. What are your views on how to strengthen the gaps?

(ત્રુટીઓ કેવી રીતે દૂર કરવિ તે અંગે તમારા મંતવ્યો શું છે?)

**Exit questions**

1. We are going to end the interview. Is there anything else you would like to say?

(અન્ય કોઈ ટિપ્પણીઓ?)
