# Supplementary material for: A mixed-methods study on impact of active case finding on pulmonary tuberculosis treatment outcomes in India
Source: Arch Public Health. 2024 Jun 20;82:92. doi: 10.1186/s13690-024-01326-0 (PMC11188491; doi:10.1186/s13690-024-01326-0)
Supplement: Supplementary file 3 — Supplementary Material 3. [file 13690_2024_1326_MOESM3_ESM.docx]

**Transcripts**

**Interview 1: Epidemiologist, 6 years’ experience**

PI: what were the aims and objectives of launching ACF as a part of TB elimination program?

C: the main objective of launching ACF in TB program was early diagnosis of the TB cases. Which will help early initiation of treatment, and thus reduce transmission and in the long run, ultimately reduce the number of cases.

PI: what is the current status of ACF in TB program?

C: ACF is a part of TB program nationally, but in Gujarat state, every 2nd Tuesday and 2nd Friday, each month, we target the high-risk areas, and followed for the whole year. Each specified day, staff goes to the site and carry out ACF activities, and if suspect any presumptive TB cases, according to symptoms, they collect the samples and then the samples are sent to the laboratory for testing.

PI: ma’am could you please share your knowledge on how much percentage of newly diagnosed cases are detected through ACF?

C: I can’t speak exactly but roughly, 2-5% of the positive TB cases are diagnosed through ACF.

PI: What do you think, can ACF affect treatment outcomes of TB?

C: I believe that the cases diagnosed early through ACF, should have better treatment outcomes. Successful outcomes should be better than PCF, that is what I believe.

PI: could you please elaborate, why do you think so?

C: I think so because there is early detection and not much of disease progression in ACF, therefore if the patient complies to the treatment, there is better curation of the disease and thus better outcomes, as compared to PCF.

PI: What are your views regarding the ACF/PCF being superior to the other?

C: I would not say superior, as both; ACF and PCF; are equally necessary for successful TB elimination program. The awareness and the education are improving, so a reasonably educated person will visit PHC for PCF on his own, so both are complimentary to each other, and not a replacement. They are both complimentary to achieve the vision of TB free india.

PI: what are the key population or the vulnerable population, we are targeting by ACF program?

C: key population includes many groups, mainly we are targeting occupational groups, like for silico-tuberculosis. Then high-risk areas like PLHA, child, close settings like jails/prisons. So, every district has a list of such key areas of their own, which are targeted by ACF.

PI: how much of contribution is by ACF in the target of eliminating TB by 2025?

C: we cannot put it in numbers or percentage, but I can use the word ‘one of the pillars’ to ultimately reduce the risk behaviour, i.e. decreasing the transmission. Basically, by early detection, we can reduce the transmission, so in a long run our goal is to decrease the number of cases, so ACF will act as one of the major pillars by using strategically.

PI: what are your views regarding intensification of TB elimination program?

C: like ACF, we are practicing latent TB prevention. Initially, if the patient developed any of the symptoms, they would approach the nearest healthcare facility, that was PCF. Then we climbed a step further with ACF, where we detect the TB cases even before the symptoms set in. and the latest strategy is to give preventive therapy to the contacts of primary TB case, who are prone to develop TB later on in foreseeable future.

**Interview 2: Senior medical officer, 11 years’ experience**

PI: what were the aims and objectives of launching ACF as a part of TB elimination program?

C: the main objective of ACF launch was early and improved detection of TB cases. Most of the patients presented late to the PHCs, after a longer duration of cough, leading to late detection. So we can avoid the late detection.

PI: what is the current status of ACF in TB program?

C: ACF has been a part of TB elimination program and its guidelines. Generally, for ACF, high-risk and vulnerable population, tribal area and urban slum areas are mapped geographically and vulnerability. These areas are surveilled bi-annually and adding to it, 2nd Tuesday and Friday of every month, at taluka level or district level, active surveillance is carried out.

PI: What do you think, can ACF affect treatment outcomes of TB?

C: rather than treatment outcomes, ACF is important for case detection. If practiced properly, ACF will obviously lead to detection of more number of TB cases, and early detection as well. Early detection will allow early initiation of treatment, and thus it can surely improve the chances of better treatment outcomes.

PI: What are your views regarding the ACF/PCF being superior to the other?

C: ideally, both are proper at their own place; ACF is also proper, PCF is also proper. But as compared to PCF, and as far as TB is concerned, ACF can help early and improved detection, otherwise, both are equally important.

PI: as you previously mentioned about the key populations, so what all key populations we target, other than you mentioned early?

C: key population will include, HIV or PLHA, diabetics, tribal population, urban slum areas, silicosis, industrial workers, cancer patients, taking chemotherapy, on steroids, post organ transplant patients, i.e. having poor immunity or taking immunosuppressant drugs are counted as key population. Over and above them, we also target de-addiction centres, where tobacco and alcohol de-addiction patients come for help, mentally ill patients; these are the population in which, TB can occur as an opportunistic infection, so these all are considered as vulnerable population.

PI: how much of contribution is by ACF in the target of eliminating TB by 2025?

C: yes, there is certain role of ACF in TB elimination. As generally, we want to decrease the incidence of TB for TB elimination goal. To decrease the incidence rate, we need to break the chain of transmission. As TB spreads through air, being an air-borne infection, we take preventive measures to prevent this air transmission, but even after that, secondary prevention is more important as compared to primary prevention where we can do early detection and prompt treatment. The main aim of ACF is early detection, so it will aid the TB elimination as it can also break the chain of transmission, which will be very beneficial to us.

PI: what are your views regarding intensification of TB elimination program?

C: ACF should be undertaken as a routine practice. Though it is a part of the guidelines, but should be done as a routine practice, especially the prisoner population should be targeted regularly. And also, to improve treatment outcomes, some patients require additional nutritional support. That should be done.

**Interview 3: Head In-charge at laboratory, 12 years’ experience**

PI: what were the aims and objectives of launching ACF as a part of TB elimination program?

C: ACF when launched by Indian govt. was an initiative to aid prime minister’s goal of eliminating TB by 2025. Usually, patients tend to use home remedy or some local cough syrup for milder symptoms, and present late to PHC or DMC, thus delaying the diagnosis. But with the help of ACF, we could do a door-to-door surveillance and detect cases at an early stage, with milder symptoms, with the help of microscopy, x-rays, and recent technology like CBNAAT. This will help early initiation of treatment and patient will soon become non-contagious. Patient can be put on treatment soon, taught cough etiquettes, and put to isolation, so he can be turned non-infectious and that was the purpose of launching ACF, along with decreasing the number of contacts with the primary case and thus overall decrease in number of new cases, which is the ultimate goal of TB elimination program, i.e. to reduce incidence rate of TB cases.

PI: what is the current status of ACF in TB program?

C: ACF is a routine practice as of now. Already the govt. of Gujarat had de-centralized the diagnostic services (DST) in 2014. Previously, the elimination of TB was a target under RNTCP program, under which, for every one lakh population, there should be a microscopic facility at DMC, but on survey, there were microscopes and lab technicians available at every PHC, however remote. So we could use those facility at PHC, at every district, every taluka. In 2019, another notice was issued by Gujarat govt. to establish a PHC wise DMC at every district. So at every district, for every suspect case, microscopy, x-ray and CBNAAT testing is done.

PI: understood. Sir could you please share your knowledge on how much percentage of newly diagnosed cases are detected through ACF?

C: actually, percentage wise data; as a microbiologist; is not known to me, that would be known better by state epidemiologist Dr. Pankaj Nimawat sir. He could guide you on this question. But the HIV positive cases, that are on ART, are offered CBNAAT testing every month. Children are offered afferent CBNAAT testing, and same for close contacts of TB also. So if the epidemiologist is able to offer you the information, you can get to know the exact percentage of the cases diagnosed by ACF. A separate record of such data is maintained by the state.

PI: as you mentioned about CBNAAT, do we subject every ACF sample collected to CBNAAT? Or some selective patients?

C: usually, it is started with microscopy only. From here, we instruct the workers that every good quality sputum sample should be subjected to CBNAAT as well as TRuNAAT. TruNAAT is available at every taluka level, around 85 machines. And around 80 machines of CBNAAT are available at district level. Both TRuNAAT and CBNAAT detects TB bacilli and Rifampicin resistance, and both are equally sensitive.

PI: What do you think, can ACF affect treatment outcomes of TB?

C: as I mentioned ago, treatment outcome data is available to the epidemiologist and he does the analysis, so he’ll be able to help you better.

PI: What are your views regarding the ACF/PCF being superior to the other?

C: when we are talking about TB elimination, none is superior to the other. When talking about TB elimination, and we have excellent diagnostic facilities, but there are limitations to every testing method, like x-rays, microscopy etc. so if we can somehow increase the diagnostic facilities and treatment of patients, we can lead to better future. We are deploying around 150 TruNAAT machines at taluka level, also pre-existing 600 DMCs are being increased to around 2200 in 2023, as compared to 2014. We have even undertook MOUs with private setups for radiological facilities, so there is early diagnosis of TB cases. We need to take aggressive steps to eliminate TB, so that the cases are diagnosed at earliest and their treatment initiated as soon as possible, we need to achieve lowest number of loss to ‘follow-up’ and decrease the failure rate by increasing patient adherence to the treatment, and thus we can achieve the dream of prime minister of decreasing incidence of TB by 2025. Aggressive approach is the key because, for example, we could isolate corona patients and increase their screening and thus decrease the spread of the disease, similarly we can diagnose TB spreader at earliest and put on treatment as soon, and thus decrease incidence. So ACF and PCF, both are to be used to full extent

PI: sir what are the key population or the vulnerable population, we are targeting by ACF program?

C: key population includes HIV positive patient, PLHA, diabetic. presumptive TB patient when comes to OPD, previous criteria was to test with cough more than 2 weeks. But current criteria includes any duration of cough, or cough with fever, or even history of weight loss are tested for sputum positivity and also offered X-ray. Wherever microscopic facilities are available, patient can effectively expectorate, we offer sputum testing. Diabetics, HIV positive, on any immunosuppressive medication, TB close contact are obviously included but we also include the regions or communities where TB prevalence is higher, there the staff is instructed to do a routine screening and especially ask and try to know about the close contact. Now the contacts are not just limited to the family members of the suspect or known case, but also his neighbours, workplace colleagues, friends or anyone. So the screening is done by analysing the prevalence of TB in a particular region and mapping is done by district TB officer and that aids in identifying the suspects. The suspects and the close contacts are also counselled about the preventive measures and about the symptoms, which if they develop, so they should contact an ASHA worker and get themselves tested at earliest. Our network/reach is widespread and can reach to most remote areas, with help of ASHA workers, male health workers, female staff etc.

PI: how much of contribution is by ACF in the target of eliminating TB by 2025?

C: WHO said 2030, but our honourable prime minister set the target of eliminating TB by 2025, before 5 years. By ACF, we have detected many cases, also detected drug resistance and also put on treatment, but out of total notified, how many are through ACF, that information is available at state level.

PI: what are your views regarding intensification of TB elimination program?

C: on direction of prime minister, central govt and state govt are already acting in this path. We are implementing new strategies as well, like we are providing nutrition support, nikshay poshan yojna, drug resistant cases are provided newer and expensive drugs like bedaquiline, delamanide, pratomanide are made available free of cost and diagnostic services are also made available. Our last prong that we can strengthen is vaccine. We should develop vaccine for adults and include it in the TB elimination program, which will aid the purpose. So vaccine will play an important role.

PI: we are about to end this interview, is there any other comments which you would like to add, please do so.

C: we are encouraging the known cases to spread awareness in his community and his surroundings. He is called a TB champion, and motivated to be DOTS provider. He can counsel other suspects, as there is a long duration of treatment for minimum of 6 months, up to 8,18 and even 24 months, and also about potential side effects of the anti-TB drugs. What he experiences, could be a great tool in counselling the other suspects and their treatment adherence. Thus, TB champion should be considered as DOT provider. Thank you

**Interview 4: City TB officer, 25 years’ experience**

PI: in your opinion, what is the current status of Active case finding (ACF) in TB program?

C: currently the status is very good, and we are covering 2700 per lakh population and we are expecting to increase the surveillance soon.

PI: what according to you is superior ACF or PCF?

C: ACF is definitely better

PI: as per you, how is ACF better or superior to PCF?

C: the cases are diagnosed early and they are diagnosed even before declared open, so that are put on appropriate treatment soon

PI: in your opinion, does ACF affect treatment outcome?

C: yes, surely. ACF leads early initiation of treatment and so better results can be obtained in patients diagnosed by ACF.

PI: in your view, how can we strengthen the gaps?

C: we need to improve and strengthen the supervision. We are training the supervisory staff, so that they can check the quality and number of sputum samples collected by the field worker. Also, the field worker needs to be trained oh how to collect the sputum? How to counsel the suspect and his family. It’ll improve the overall outcome of the program.

PI: would you like to share any other views on how to intensify TB elimination target by 2025?

C: we are focusing on the predictive TB cases, and attention to those children who have not gained sufficient weight in past 2 months or have lost weight are subjected to testing and chest X-ray.

**Interview 5: WHO national consultant, 9 years’ experience**

PI: what were the aims and objectives of launching ACF as a part of TB elimination program?

C: so basically, what is idea behind the ACF is that; for any case, there are two components, passive case finding and active case finding, that is actively going in the community and searching for the cases. So previously we highly relied on PCF, the person who are coming to the health facility having the sign and symptoms of TB, that are screened. In this methodology, large number of cases were missed in the diagnosis. As well as there is delay in the diagnosis. There are research studies that show that the patients getting diagnosed, may likely to get delayed upto 15-20 days as well as they visit around 1-7 facilities before actually diagnosing the TB cases. So, this ACF model is a good implementation to have an early case detection and that is why we are going house-to-house to detect cases early. So overall, ACF is great tool to detect those missing cases, as well as kind of early case detection.

PI: what is the current status of ACF in TB program?

C: I don’t have status check, even TB cell would not have exact percentage of cases detected through ACF, from total cases detected. But if I roughly estimate, around 5-7% of the total cases are detected through ACF. But this is just an estimate. Early detection of cases is much more important than the proportion of the additional yield through ACF. The cases which are going to be missed, we are picking up in an early stage, to be put on apt regimen and Treatment

PI: What do you think, can ACF affect treatment outcomes of TB?

C: what I understood and realize, where there is an ACF, if there is an element of doing proper ACF, then it would have very good impact in the Treatment outcome. We need a sensitive tool to rule out TB in the community first of all. Screening tool needs to be highly sensitive. In ACF, what I realize is, large no. of cases detected in community are presumptive TB cases and are based on symptoms only, very few cases are offered CHEST X-RAY. If screening tool is highly sensitive, if symptoms are topped with CHEST X-RAY, then it is highly likely that case won’t be missed and the same time if detected early would improve Treatment outcome. But I would not say that there will be any impact or difference in case diagnosed through ACF vs. PCF. Even what I realize is Treatment outcome will be better in PCF, that is what I assume.

PI: would you like to elaborate why do you think so?

C: because, first of all, when any case is diagnosed by PCF, it is run thorough number of investigations. So the cases that have willingness to conduct series of testing, as well as going into the facility with the notion that they’ll be taking the medicines. They require the assistance of the doctor for itt. With that mindset of patient. so patient identified by PCF will be more acceptable of the treatment and the long course of 6 months, so that’s why I think that PCF will be better Treatment outcomes.

PI: sir what are the key population or the vulnerable population, we are targeting by ACF program?

C: there is a standard guideline published by GoI, in which they have highlighted the 26 vulnerable population. It includes prisoners, silicosis, dialysis, immunosuppressive therapy, even urban slums and tribal populations. So basically these are the high risk grp for TB. It is largely stratified into 3 groups, clinically vulnerable, socially vulnerable and marginalized population.

PI: what are your views regarding intensification of TB elimination program?

C: it has an impact of, first of all, identification of missed cases and secondly early detection and Treatment, that’ll create an impact in the case finding and secondary prevention, is one of the strategies, we can reduce the vulnerability and infectiousness in the community.

PI: in your view, how can we strengthen the gaps?

C: firstly, for the ACF: current major challenge is to have screening tool. We recently completed TB prevalence survey, which taught us that for case detection, if 100 cases are detected by only symptoms and not complimented with CHEST X-RAY, the sensitivity is only 50%. Thus, we miss 50% of patients, which some people call it subclinical TB case. Hence we need highly sensitive tool for apt yield and coverage. Planning is supposed to be very meticulous for detecting the cases. There are tools available which can give idea where u need to conduct ACF. We all know the beneficial effect of ACF in early case detection. Our aim is not only increasing the number of case detection, but also parallelly rule out TB in the same community. Those ruled out, should be put on preventive Treatment, by checking for latent tb infection positivity. So the objective of the intervention is not only case detection, but also prevent latent tb infection.

**Interview 6: senior treatment supervisor, 20 years’ experience**

PI: in your opinion, what is the current status of Active case finding (ACF) in TB program?

C: talking about urban areas, ACF is stronger as compared to rural areas, because of the deficit of sufficient working staff, which is only government healthcare workers, whereas in urban, there is better cooperation between corporation and government healthcare workers, so there are better results in urban, and not so much in rural

PI: overall, what is the status of ACF in Bhavnagar district?

C: it is good, overall urban and rural, both are good

PI: we practice both ACF and PCF for TB, what according to you is better?

C: ACF, because it aids our short-term goal of eliminating TB by 2025, whereas PCF is far fetched approach for elimination of TB

PI: as you said, ACF is better, could you please elaborate as how is ACF better than PCF, for what reasons is ACF better?

C: there are a few reasons as to how ACF is better. Firstly, because of scheduled approach, there is increased awareness in the common public, they are well-aware that healthcare workers visit their community on 2nd and 4th Tuesday of every month, so that is advantageous. Also, due to scheduling of the visits, the healthcare staff can also organize and plan their strategies.

PI: anything else, you’d like to add to the list of pros of ACF?

C: yes, recently, government has started incentive of Rs. 25 per sample, for all ASHA workers, that motivates them to work more efficiently.

PI: okay sir, understood. What is your opinion on the importance of ACF in improving treatment outcomes, as contrast to PCF?

C: as such, there is no role of ACF in improving treatment outcomes, but there is surely increased and more effective case finding from close contacts, due to tracing of the primary ACF case, and also increased awareness in the public. But there is no improvement in treatment outcomes.

PI: our hypothesis is that, if ACF helps in early diagnosis, then there is early initiation of treatment, if positive, and thus better treatment outcomes, even before noticeable symptoms appear.

C: yes, definitely there is early initiation of treatment, but no difference in treatment outcome in comparison to PCF. On the contrary, there is a small 0.5% chance of case turning into a defaulter, due to spot sample being ‘scanty’ in the diagnosis chain.

PI: what are your views on how to strengthen the gaps?

C: it is a matter of innovation. We should try to make the diagnosis errorless. We have CBNAAT and TruNAAT, which are excellent diagnostic tools for diagnosis as well as for detecting resistance, but even they fail when the bacterial load is scanty. So if could develop some technology to solve that, it would be great.

Also, we’ve recently started incentive for the patients of TB. If they send any likely suspect for analysis, they receive an incentive of Rs. 25/suspect.

PI: it was an interesting conversation, if there is anything that you would like to add to the conversation regarding ACF and/or PCF, please do so.

C: currently, ACF is practiced only twice a month, which should be increased to four times a month.

And also, we should indulge into constructive punishment for healthcare workers involved in this program, for their negligence and soldering on their job.

For example, I punished all the 8 teams for their lack of efficiency, when they did not collect any samples. I ordered them to visit again the next day, and collect samples. They collected around 25 samples, out of which 2 turned out to be positive. So, such practices will help.

**Interview 7: District TB officer, 20 years’ experience**

PI: What according to you is the status of ACF in TB program?

C: frontline workers conduct a door-to-door surveillance and try to collect samples from the suspects, and they are followed up if positive.

PI: What are your views regarding the ACF/PCF being superior to the other?

C: ACF should be considered superior, as public will visit nearby hospital if they are symptomatic, but they tend to ignore minor ailments, and thus by early detection by ACF, it should be considered superior. But in reality, we don’t observe this difference due lack of public participation.

PI: What do you think, can ACF/PCF affect treatment outcomes of TB?

C: we do not receive many cases through ACF, so it’d be difficult to comment on its effect on treatment outcomes. We can surely hypothesize that due to early initiation of treatment, there should be improvement in treatment outcome but unfortunately, we do not have sufficient evidence or data to back it up.

PI: sir, what are your views, as how to strengthen the gaps in this ACF program?

C: ACF surveillance is carried out by general health-staff, which is also occupied in other programs or works, so it is not their sole priority, hence we are not receiving the expected outcome of the ACF program right now, but it work in progress, so we are improving it day by day.

PI: would you like to share any other views on how to intensify TB elimination target by 2025?

C: public awareness is a crucial step in this process. Patient should be educated on self-screening and on the preventive measures and the prognosis of this disease. At community level, still there is not much awareness seen regarding this disease. There’s already being a lot done by the government, but until the public is made aware, we cannot achieve this goal.

PI: is there anything that you’d like to add to this conversation, before we end this?

C: as said, there’s lack of dedicated staff for ACF surveillance, which if solved, could make a good difference. Also, if NGO’s get involved and their funds can be utilized to incentivize the patients, that’d help our purpose.

**Interview 8: Nodal officer cum medical officer, 12 years’ experience**

PI: What were the aims/objectives of the ACF programme that was launched or why was it launched?

C: ACF was launched because earlier it was observed that screening only occoured of the cases that presented in the opd. So many high risk areas were left out and the people there did not have access to medical facilities because of many reasons like distance, time in case of employed people, etc. so for such groups of people ACF programme was launched by the state. Before that Chhattisgarh was the only state that launched such a programme and received positive feedback and hence, this was introduced in the whole country. And because Gujarat is such an active state they started ACF programme in high risk population in labourers, people working in industries like gidc, health care workers and areas where 2+ or 3+ cases were found in the last 2 years were identified and ACF was done there which yielded positive results because of early identification. Sputum collection of such people was done from their houses only by the health care workers like ASHA and were taken to the nearby health facilities like phcs and bmc( all phcs are bmc after 2016) and were examined. These tests found many positive cases and such patients were informed and early treatment was started and so the outcome of such patients was much better.

PI: What is the current status of ACF in the tb programme?

C: If 100 cases of tb are diagnosed then 5% of cases are recovered now which was around 2% in 2014-15 and in 2019-20, the results reached 5-6% which means of of 100 cases identified, 5 cases were by ACF.

PI: Out of ACF and PCF which one is superior or better?

C: The aim of any health organisation is to improve health of the people or to make everyone healthy. And because prevention is better than cure and also field visits are done after every 15 days by our health care workers. So the patients found with symptoms early and their treatment can be better and started earlier which meant good prognosis. It is well known that tb affects the adult population which is from 15-45 years of age because they have to stay out of their homes for various reasons and so are easily infected. So if screening is done through ACF in such population like bd population, people working in gidc, densely populated areas or even in health care workers, early detection of tb cases is possible. Such people are given prompt treatment and are cured very easily. So treatment is possible before it is too late so according to me, ACF is better than PCF.

**Interview 9: WHO consultant, 5 years’ experience**

PI: what were the aims and objectives of launching ACF as a part of TB elimination program?

C: the main objective of ACF was to identify missing TB cases and early detection.

PI: what is the current status of ACF in TB program?

C: in Gujarat, ACF is practiced regularly on every 2nd Tuesday and Friday of each month

PI: What do you think, can ACF affect treatment outcomes of TB?

C: ACF leads to early diagnosis and thus, pt can be put on appropriate treatment outcome at the earliest, so it can definitely improve treatment outcomes of TB patients.

PI: What are your views regarding the ACF/PCF being superior to the other?

C: in my opinion, PCF is superior to ACF, because it is more sustainable and more acceptable than ACF. With PCF, pt comes to hospital with seeking treatment, while in ACF, pt is relatively healthy and asked to be put on treatment for such long duration. This decreases the pt acceptability and compliance. In PCF, there is need limited of resources and staff as there is a single facility, while in ACF, we require large number of staff

PI: what are the key population or the vulnerable population, we are targeting by ACF program?

C: the key populations include HIV positives, silicosis pt, diabetics, immunocompromised pt etc.

PI: how much of contribution is by ACF in the target of eliminating TB by 2025?

C: we should expand our search for ACF to general population as well, and not just key population, to include any missed cases. Secondly, we should rationalize the resources available, so we can exploit them strategically. Thirdly, strategy of ring survey should be implied. Past case history should be taken very extensively, to know for latent TB infection. Lastly, yield should be increased to increase the rate of identifying new cases and possibly include any missed cases.

PI: what are your views regarding intensification of TB elimination program?

C: we should implement DST guidelines and focus on TPT (TB preventive therapy) for the close contacts and to prevent LTBI. This will largely reduce the incidence. Improve and initiate vaccination program even for adults, which can effectively reduce the incidence as well.

PI: we are about to end this interview, is there any other comments which you would like to add, please do so.

C: I feel that there is no strict control over private sector, which should be initiated, so that medicines are available to the patients at subsidized rates and so there is no discrimination. Even drug resistant TB medicines to be made available to every applicable patient. This will reduce the prevalence of DR-TB and also help in elimination of TB by 2025.

**Interview 10: Additional director at STDC, 15 years’ experience**

PI: what were the aims and objectives of launching ACF as a part of TB elimination program?

C: when new technical operational guidelines were launched in 2017 nationwide, before that, in Gujarat, rapar taluka of bhachau district was the first to start this ACF activities. House to house survey was conducted, and symptomatically screened. And those who were symptomatic, were subjected to sputum analysis and CXR, and diagnosed further. What started in rapar taluka, was nationwide implemented.

PI: what is the current status of ACF in TB program? And what percentage of total cases are diagnosed through ACF?

C: talking about percentage, around 2-3% positivity is through ACF, but basic benefit of ACF is early detection. In PCF, when the pt comes to us, he might already be bedridden or sputum 2+/3+ or 2-3 months of symptoms. But in ACF, house to house survey helps in early detection of these cases, especially the close contacts and the high risk groups, in which there is higher chances of infection as compared to general population. But early detection is the advantage of this.

PI: what is the contribution of ACF in the program?

C: there are a lot of advantages to the program. Early detection will help put the pt on prompt Treatment and increase the rate of sputum conversion, i.e if the pt is detected early when he is scanty/+1, if put on Treatment, will turn non infectious as compared to when the pt visits at 3+ . additionally, those missed usually, when subjected to CXR, during ACF activities, are also detected. Also, we subject the samples collected through ACF to CBNAAT, which is more sensitive to sputum microscopy, which can also improve Treatment outcomes.

PI: is CBNAAT practiced only for ACF?

C: no it is a part of universal DST, and additionally, for the mucopurulent samples collected from key population. It has high sensitivity of around 90%. It increases the yield.

PI: what are the key population that we target?

C: urban slums, diabetics, immunocompromised, malnourished, antenatal populations are the primary target… on 2nd and 4th Tuesday of the month. In tribal, there is higher number of incidences.

PI: What do you think, can ACF affect treatment outcomes of TB?

C: sputum conversion, compliance, co-morbidities.. improved outcomes.. and early diagnosis will help in early cure of disease and thus reduce transmission.

PI: according to you, in TB elimination by 2025, what is the contribution of intensified ACF?

C: focus on vulnerable pop. Identify hot spots and cold spots, which is possible with taluka-wise mapping or district-wise mapping. Intensify at hot spots. And use the concept of ‘TB-free village’ at cold-spots, and strengthen ACF to general population of that area.

PI: what is your opinion on; will TB be eliminated by 2025?

C: if put of Treatment immediately, chain of transmission is broken, there are definitely better Treatment outcomes. And there is progress in development of vaccine, cough etiquettes, TPT, universal DST, and then apt drugs made available.. everything in total will help… current decrease in incidence is around 2.5%, but it should be reached to 10%, to successfully eliminate TB. All of these is necessary for it.

PI: would you like to suggest any changes or interventions under NTEP?

C: focus on 3 things… 1. Vaccine 2. TB preventive therapy.. current prevalence of infection is 30%. Treatment, if made available for LTBI, them turning into active case is prevented, thirdly, mapping… hot spots.. cold spots.. TB free village certified, if detected in those area, so ring survey, and block the transmission. Ring TPT by testing close neighbours and contacts for IGRA, give TPT and thus break chain.

PI: can you please elaborate about the vaccine, that you mentioned?

C: currently, we only have BCG vaccine for children, but we need to develop a vaccine for adults as well. With good efficacy and long duration of prevention.

PI: anything you’d like to add to your statement?

C: develop soft skills, by the surveillance staff. train them to identify maximum number of cases in their respective areas. Map under NIKSHAY. Survey them, screen them, treat them and thus, the spread is stopped. Supervision and monitoring of the staff are a must. Xray and CBNAAT for ALL sputum should be made must.

PI: do we have all the resources and logistics?

C: CBNAAT and truNAAT will be centralized; we have 306 units already, of which 150 are CBNAAT.. increase to all units.. point of care test.. Diagnosis at the same place as symptom screening and DST facility.

**Interview 11: WHO consultant, 2 years’ experience**

PI: what were the aims and objectives of launching ACF as a part of TB elimination program?

C: as per my knowledge, we have nearly 50% health seeking behaviour in population; which is very low, if we look in regards of problem, so due to lack of this, we were missing many cases which were symptomatic and still undiagnosed. So, in order to reduce this numbers of patients and to enhance health seeking behaviour, ACF is better approach. Secondly, we can get better yield by focusing on vulnerable groups, which is not possible in PCF. Factors like socioeconomic class and geographic representations cannot be studied through PCF, which is also possible in ACF. So, these were the objectives of launching ACF program.

PI: what is the current status of ACF in TB program?

C: very less, around 2% or less than that. Because we do ACF but we still need to work on quality of the process. Because we find nearly 1000 cases only through ACF while our total notification is around 1.5 lakh per state.

PI: could you please share your knowledge on how much percentage of newly diagnosed cases are detected through ACF?

C: as I said before, it comes nearly 2%, if we calculate

PI: What do you think, can ACF affect treatment outcomes of TB?

C: it can enhance treatment outcome because we can find cases at and earlier stages through ACF.

PI: What are your views regarding the ACF/PCF being superior to the other?

C: actually, we cannot compare both of them. Because ACF is focused on particular vulnerable groups only, while PCF is for general population. So, they are not alternatives, they need to be done together. But if you want to focus vulnerable group, ACF is better opinion.

PI: sir what are the key population or the vulnerable population, we are targeting by ACF program?

C: as mentioned in NIKSHAY module and guidelines, we focus on comorbidity like diabetes, hypertension, age group above 60, some particular geographic areas like we cover all tribal population in respective areas, prisoners, malnourished cases, migrant population, industrial workers etc.

PI: how much of contribution is by measures other than ACF in the target of eliminating TB by 2025?

C: currently, we are in trial stage of vaccination for disease prevention as you know BCG does not prevent but decreases severity only. But until the vaccine is available, we have to take measures to prevent infection spread and we have many such regimens available for same purpose and early detection of case also plays greater role in good outcomes, as it also prevents spreading due to start of treatment earlier which leads to decrease in secondary cases. Also, proper treatment adherence, are monitoring is a key to good outcome, generally pulmonary cases become sample negative in 7-15 days if adhered to regimen and monitored properly.

PI: what are your views regarding intensification of TB elimination program?

C: intensification is possible if we do monitoring from planning to execution level thoroughly as for example, when we do mapping, it should be decentralised like every PHC should be identifying their own vulnerable group by themselves, but many-a-times mapping only takes place at district or higher centre only. So, we miss vulnerable groups which should get detected during mapping. Secondly, due to lack of sensitivity of our team, we are able to find only less than 1% of presenting cases instead of 3-5%, which is ideal situation. For an example, due to lack of participation or improper sensitisation of team which leads to difficulty in convincing population for sputum collection or sometimes we compromise with quality of collected sputum sample.
