# Supplementary material for: A mixed-methods study on impact of active case finding on pulmonary tuberculosis treatment outcomes in India
Source: Arch Public Health. 2024 Jun 20;82:92. doi: 10.1186/s13690-024-01326-0 (PMC11188491; doi:10.1186/s13690-024-01326-0)
Supplement: Supplementary file 4 — Supplementary Material 4. [file 13690_2024_1326_MOESM4_ESM.docx]

**Supplementary Table 1: Description of codes on the themes ‘ACF and TB outcomes’ and ‘Strengthening ACF implementation’ as perceived by the TB program functionaries during January-April 2023 in Gujarat**

| **Themes** | **Categories** | **Codes** | **Description** |
| --- | --- | --- | --- |
| ACF and TB outcomes | ACF objectives | TB elimination | ACF was a new intervention launched under NTEP that will help eliminate TB. |
|  |  | Timely diagnosis and treatment | Early diagnosis of TB cases will help initiating prompt treatments sooner. |
|  |  | Break transmission chain | Sooner the treatment initiated, sooner will the patient turn non-infectious, and thus reduce the spread. |
|  |  | Detecting missed cases | Screening of vulnerable population, backward tribes, and household contacts, will reduce the cases that would have been missed otherwise. |
|  |  | Increase yield | Detecting missed cases and screening household contacts will help increase yield. |
|  | ACF superiority | Reduced unfavourable outcomes | Early detection and prompt initiation of treatment will prevent unfavourable treatment outcomes. |
|  |  | Early sputum conversion | Sooner the treatment initiated, sooner will the patient turn non-infectious, and thus sooner the sputum conversion. |
|  |  | Increases awareness | Conducting survey in community will increase patient awareness and education regarding cough etiquettes, symptoms of TB and its sequalae. |
|  |  | Rules out TB | A negative screening will rule out TB in asymptomatic cases. |
|  |  | Reduced sequelae | Early initiation of treatment will prevent development of TB complications, which could happen if untreated or diagnosed at late stages. |
|  |  | Reduce incidence | Rendering primary case non-infectious at early stage will ultimately reduce the incidence. |
|  | PCF superiority | Improved compliance | As the patient are symptomatic as the point of diagnosis, patient is more likely to comply to the long-term regimen. |
|  |  | Treatment seeking behaviour | When a symptomatic case visits hospital by their own, they tend to seek treatment and thus more likely to comply. |
| Strengthening ACF implementation | ACF in NTEP | Increase mapping | ACF staff should analyse the survey and map high-prevalence spots in their region. |
|  |  | Prevalence estimation | ACF helps in systematic assessment of TB prevalence in a population through proactive case detection methods. |
|  | Strengthening ACF | More visits | Currently the survey is conducted twice a week, but increasing the frequency will aid increase yield. |
|  |  | Dedicated staff | Currently, general survey staff conducts ACF surveillance, but a dedicated staff for ACF will result in better and quicker achievement of goals. |
|  |  | General population coverage | Vulnerable population are screened right now, which should to expanded to general population as well, so that missed cases can be detected. |
|  |  | Incentivise ASHA | Incentivising ASHA workers for each suspect they bring to screening, will provide motivation, and ultimately increase yield. |
|  |  | Increase supervision | A dedicated officer appointed to overview and supervise the ACF surveillance, for strict vigilance will improve conductance of the program. |
|  | Strengthening NTEP | Better diagnostic tests | Availability of newer and more sensitive diagnostic tests be made available at every DMC. |
|  |  | Patient awareness and education | Special dedicated programs be done at community level to educate the general population and increase awareness. |
|  |  | Ring surveys | Ring survey implies that the close contacts of the primary case should be traced and screened. |
|  |  | Resource rationalization | Due to resource poor setting, we must rationalise the available resources to fully exploit them to our best advantage. |

ACF: Active Case Finding, ASHA: Accredited Social Health Activist, DMC: Designated Microscopy Center, NTEP: National Tuberculosis Elimination Program, TB: Tuberculosis,
