# Supplementary figures and images for: A mixed-methods study on impact of active case finding on pulmonary tuberculosis treatment outcomes in India
Source: Arch Public Health. 2024 Jun 20;82:92. doi: 10.1186/s13690-024-01326-0 (PMC11188491; doi:10.1186/s13690-024-01326-0)

Intra-cluster correlation coefficient (ICC) output


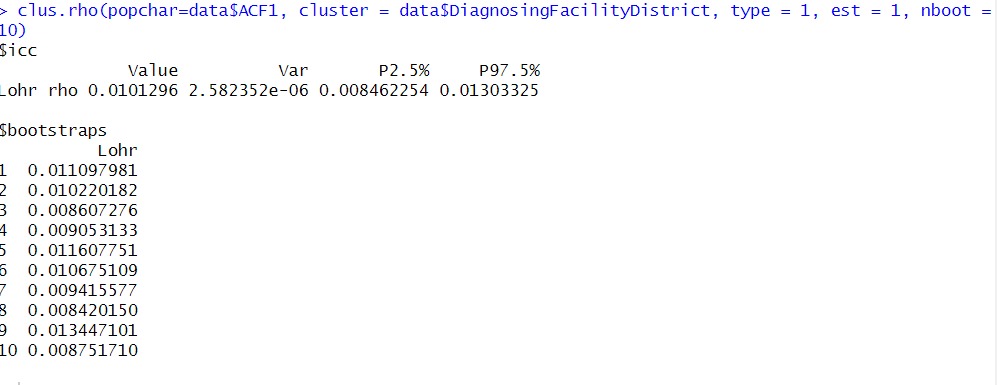


Bootstrap analysis


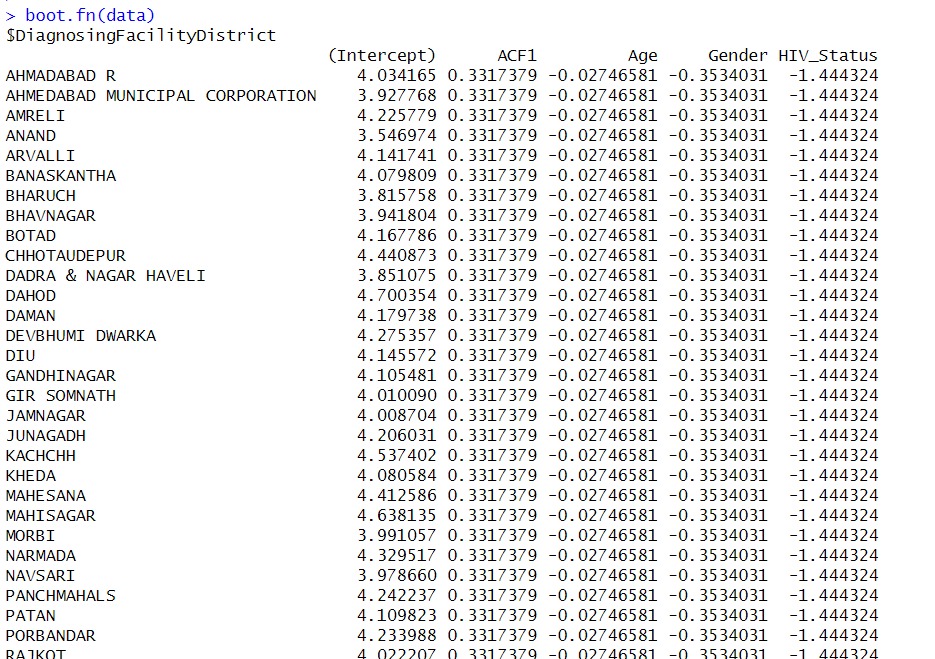

Supplement: Supplementary file 5 — Supplementary Material 5. [file 13690_2024_1326_MOESM5_ESM.docx]
